# Bacterial endocarditis caused by contact lens usage

**DOI:** 10.1186/s44215-024-00134-w

**Published:** 2024-03-08

**Authors:** Susumu Ishikawa, Hiroki Matsunaga, Hideki Mishima, Yasushi Katayama, Koichi Yuri, Koichi Ohashi, Daisuke Abe

**Affiliations:** 1 Department of Thoracic and Cardiovascular Surgery, Tokyo Metropolitan Bokutoh Hospital, 4-23-15 Kotobashi, Sumida-ku, Tokyo, 130-8575 Japan; 2 Department of Cardiology, Tokyo Metropolitan Bokutoh Hospital, Tokyo, Japan

**Keywords:** Infective endocarditis, Contact lens, Mitral valve surgery

## Abstract

A 17-year-old female was transferred to our hospital due to high fever, general fatigue, and dim eyesight. Three weeks before, she had used cosmetic colored contact lenses and then suffered from bloodshot eyes associated with dim eyesight. Intermittent fever and general fatigue were followed by eye symptoms. Echocardiography revealed moving vegetation on the posterior leaflet of the mitral valve associated with mild mitral valve regurgitation. There were no infectious sites in systemic examinations; thus, the cause of infective endocarditis was considered the infection due to contact lens usage. The patient initially received mitral valve plasty associated with the removal of infective sites. However, redo surgery was necessary 19 days later due to the relapse of infection, and the mitral valve was replaced by bioprosthesis. Traumatic injury of vessels due to inappropriate contact lens usage seemed to lead to systemic hematogenous infection and subsequent endocarditis. We report a rare case of infective endocarditis which was caused by contact lens usage.

## Introduction

Bacterial endocarditis is still a serious condition associated with non-neglectable morbidity and high mortality. Various causes of infective endocarditis are reported such as oral infection, urinary infection, pneumonia, and so on. However, keratitis due to contact lens usage has not been reported as the cause of infective endocarditis. We report a rare case of infective endocarditis in a young female.

## Case presentation

A 17-year-old female was transferred to our hospital due to high fever, general fatigue, and dim eyesight. Three weeks before, she had used cosmetic colored contact lenses which were bought at the supermarket, and then suffered from bloodshot eyes associated with dim eyesight. Intermittent fever and general fatigue also were followed by eye symptoms. Medication of oral antibacterial and antipyretic agents was given by a neighboring doctor, but the fever was sustained.

On hospital arrival, systemic blood pressure was maintained and associated with sinus tachycardia. No chest murmur, strider, or extremity edema was observed. Osler’s nodes were detected in bilateral palms and soles of feet. Subungual hemorrhage were observed in the nail beds of bilateral thumbs. Bleeding spots existed on bilateral bulbar conjunctivas.

A slit lamp examination found multiple hemorrhages on bilateral eyegrounds with Roth’s spots.

Hemorrhage on the eyeground extended to macula in the right eye and it was considered the cause of eyesight failure. However, endophthalmitis, a severe intraocular infection was not observed.

In blood analysis, values of white blood cell count (20400/μl) and C-reactive protein value (23 mg/dl) were highly elevated, and platelet cell count decreased (41,000/μl). Transthoracic and transesophageal echocardiography revealed mild mitral valve regurgitation. Left ventricular ejection function was preserved (63%) and the aortic valve was normal. Vegetation was not apparent at the initial examination on admission, however just before surgery a moving vegetation (10 × 12 mm in size) was detected on the posterior leaflet of the mitral valve. There were no infectious sites in systemic examinations including oral, brain, lung, abdomen, genitourinary tracts, or skin lesions. Thus, the cause of infective endocarditis was considered the infection due to contact lens usage.

In the bacterial culture of blood samples, methicillin-susceptible Staphylococcus aureus (MSSA) was detected. Cefepime dihydrochloride hydrate (CFPM) and Vancomycin (VCM) were used as antibacterial agents. In spite of the intravenous infusion of antibacterial agents, white blood cell count, and C-reactive protein level remained high (24300/μl and 18 mg/dl, respectively). Semi-emergency operation was performed on the 4th day of admission especially considering the moving vegetation on the mitral valve.

**Fig. 1:**
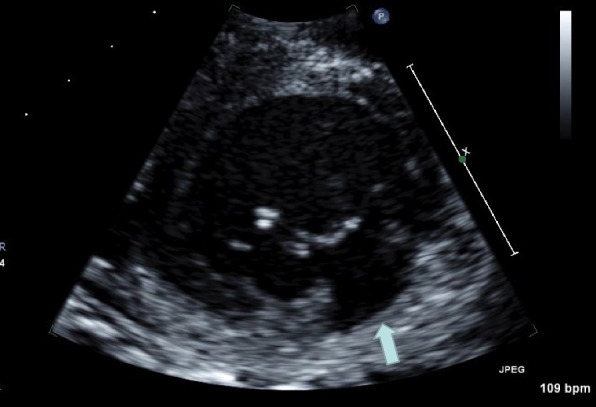
Transthoracic echocardiography before the second surgery. The shorting of the posterior leaflet is observed especially on the median side (arrow)

A median sternotomy with a shorter skin incision was performed and the mitral valve was approached through atrial septal incision under cardiac arrest. Massive vegetation attached mainly on the posterior leaflet of the mitral valve and sub-valvular structures, which were carefully removed. The posterior mitral valve leaflet was hyperplasic, however, not destroyed. Thus, we preserved a metal valve with two plastic stitches on the small defect of the posterior leaflet which was resected with attached vegetation. After the surgical field was irrigated with a saline solution, 0.1% Gentian Violet solution was applied to the mitral valve leaflets and sub-valvular structures. Mild mitral valve regurgitation was observed by transesophageal echocardiography at the end of surgery. Bacterial examination of resected vegetation revealed MSSA. Just after surgery, her circulatory and respiratory conditions were stable and blood examination results gradually recovered. Blood cultures were negative. However, cardiac failure and the relapse of infection appeared on the 10th day after surgery. Transthoracic echocardiography revealed severe mitral valve regurgitation associated with the shorting of the posterior leaflet especially on the median side (Fig. 1).

**Fig. 2:**
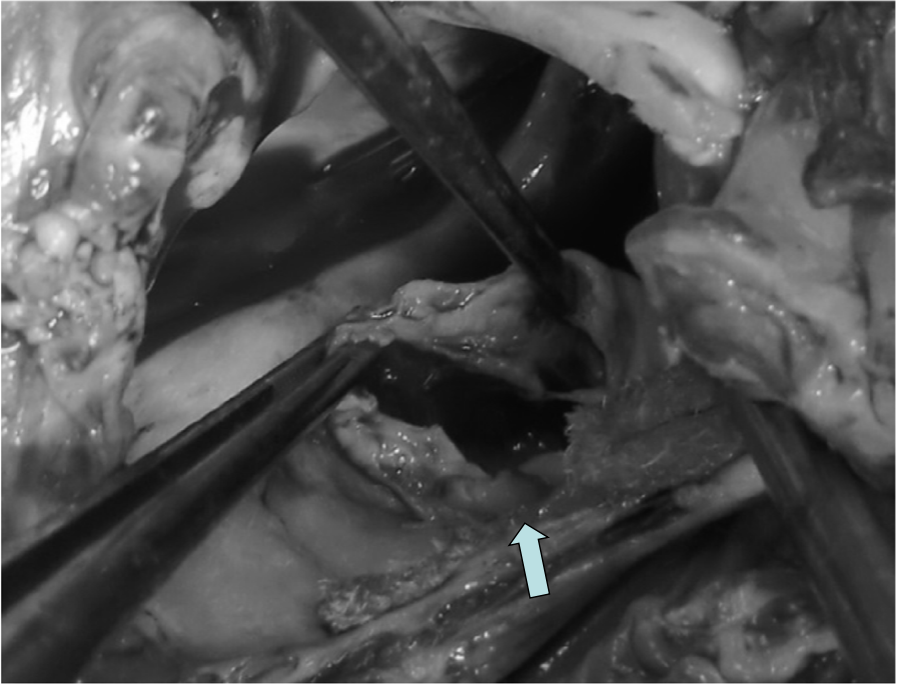
Operative findings of the mitral valve during the second surgery. Two-thirds of the posterior leaflet and sub-valvular structures almost disappeared in the median side of the mitral valve (arrow)

Redo surgery was performed on the 19th day after the initial operation. The chest was reopened along the previous incision and the mitral valve was approached through the atrial septal incision. Two-thirds of the posterior leaflet and sub-valvular structures almost disappeared in the median side of the mitral valve (Fig. 2). The anterior leaflet and lateral side of the posterior leaflet were not infected visually and preserved. The mitral valve was replaced with Carpentier-Edwards bioprosthesis (23 mm in diameter). The postoperative course was uneventful. Three years after surgery, transthoracic echocardiography revealed no evidence of re-infection and CRP values stayed normal.

## Discussion

Infective keratitis in contact lens users was a common disease. Teo L, et al. [1] report infective keratitis was seen in 243 (25.6%) out of 953 contact lens users, with 55 patients requiring hospital admission for management of the infection.

Microbial keratitis in contact lens wear is more common in young patients and is more often caused by Pseudomonas aeruginosa and Acanthamoeba spp. Thus, a trial for limiting the risk of infection associated with decorative or cosmetic contact lens wear is recommended [2]. Our reported case was not familiar with the sterile management of contact lenses because she had good eyesight and no experience of using contact lenses or glasses.

Among eye diseases, endophthalmitis is sometimes observed as the first manifestation of infective endocarditis [3, 4]. Endophthalmitis is also reported as the cause of systemic infections such as endocarditis [5]. Hsiang-Chun Lee, et al. report a rare infective endocarditis case caused by uveitis [6]. In our case, there were no endophthalmitis or uveitis. Thus, the cause of infective endocarditis was considered keratitis due to contact lens usage. The palpebral conjunctiva is rich in capillary vessels and hordeolum was frequently caused by Staphylococcus aureus [7, 8], which was detected in our case. Traumatic vessel injury of palpebral conjunctiva due to inappropriate contact lens usage seemed to lead to systemic hematogenous MSSA infection and subsequent endocarditis.

The results of eye discharge culture might be the most important evidence for of infective route. However, we did not hit upon that eye disease was the cause of systemic infection at the time of the initial emergency examination. We thought that eye symptoms were the effects of systemic infection. Thus, we did not collect eye discharge culture but only blood culture. After confirming the results of systemic examinations and bacterial culture of blood samples, we finally confirmed the cause of infective endocarditis was the infection due to inappropriate contact lens usage.

Generally, the operative results of active IE are worse than those of healed IE.

A complication of the recent cerebrovascular accident is reported as an independent predictor of mortality in active IE [9]. Doukas G, et al. [10] report operative mortality was 2.8% in 36 patients with active culture-positive infective MV endocarditis.

Early surgery for IE has been advocated in general. Urgent surgical treatment for IE is recommended when signs of uncontrolled infection are present, including abscess formation, fistula formation, enlarging vegetation, and pseudoaneurysm formation [11].

In our case, early surgery was selected due to uncontrolled infection and enlarging vegetation.

In aspects of surgical procedures, mitral valve repair is recommended even for active culture-positive infective endocarditis [10, 12]. In the initial surgery, we aimed for sufficient removal of vegetation and valve repair. However, infectious sites remained probably in the posterior mitral valve leaflet which were visually considered as hyperplastic. In the second surgery, the mitral valve was replaced with bioprosthesis considering that the patient was a young female. The postoperative course and follow-up results were uneventful. We reported a rare case of infective endocarditis caused by contact lens usage.

## Conclusion

We reported a rare case of infective endocarditis caused by contact lens usage. Traumatic vessel injury of palpebral conjunctiva due to inappropriate contact lens usage seemed to lead to systemic hematogenous infection and subsequent endocarditis.

## Data Availability

Written informed consent was obtained from the patient for publication of this case report and accompanying images.
